# Valorization of Tomato Seed By-Products as a Source of Fatty Acids and Bioactive Compounds by Using Advanced Extraction Techniques

**DOI:** 10.3390/foods11162408

**Published:** 2022-08-11

**Authors:** Ignacio Solaberrieta, Ana Cristina Mellinas, Jérémy Espagnol, Mahmoud Hamzaoui, Alfonso Jiménez, María Carmen Garrigós

**Affiliations:** 1 Department of Analytical Chemistry, Nutrition & Food Sciences, University of Alicante, San Vicente del Raspeig, ES-03690 Alicante, Spain; 2 Biomass Valorisation Platform, Celabor s.c.r.l., Avenue du Parc 38, 4650 Herve, Belgium

**Keywords:** tomato seed by-products, waste valorization, microwave-assisted extraction, supercritical fluid extraction, fatty acid methyl esters, tocopherols, antioxidant activity

## Abstract

In this work, lipids and bioactive compounds from tomato seed by-products were extracted and compared by using advanced extraction techniques, such as microwave-assisted extraction (MAE) and supercritical fluid extraction (SFE). The influence of different extraction parameters, including extraction temperature (T), time (t) and solvent volume (V) for MAE as well as extraction temperature (T), pressure (P) and flow rate (F) for SFE-CO_2_, was evaluated on tomato seed oil (TSO) yield and fatty acids composition using response surface methodology (RSM). Optimum extraction conditions for MAE were 56.2 °C, 29.0 min, and 67.6 mL, whereas conditions of 60.2 °C, 400.0 bar, and 64.6 g min^−1^ were found for SFE-CO_2_. Under these conditions, higher TSO extraction yields were obtained by MAE compared to SFE-CO_2_ (25.3 wt% and 16.9 wt%, respectively), while similar fatty acids profiles were found by GC in terms of FAMEs composition: methyl palmitate, methyl stearate, methyl oleate, and methyl linoleate, accounting for around 80 wt% of unsaturated fatty acids. TSO MAE extracts showed high DPPH• radical scavenging activity which was related to the presence of tocopherols; in particular γ-tocopherol, which was found as the dominant homologue (260.3 ± 0.6 mg kg_TS_^−1^) followed by a lower amount of α-tocopherol (6.53 ± 0.12 mg kg_TS_^−1^) by HPLC-DAD. The obtained results suggested that tomato seeds are an interesting source of bioactive compounds with potential use in a wide range of nutritional and food applications, increasing the added value of this by-product, which is currently underexploited.

## 1. Introduction

Tomato stands out as one of the most widely produced and consumed vegetables in the world, only after potato. It is rich in beneficial components such as carotenoids, phenolics, and vitamins, among others [1,2,3], of which the intake in human diet has been related to protective effects towards cardiovascular diseases, cancers, and oxidative stress [4]. Even though a relatively small fraction of tomato is consumed as a fresh vegetable, most of the global production is processed into several products, such as paste, puree, sauce, and ketchup [4,5,6,7]. According to FAO, the global tomato production and the soil area destined to its cultivars are continuously rising [5]. Tomato harvesting and processing generate a considerable amount of waste which is mainly composed of peels and seeds, constituting a serious environmental and economic problem. Recent studies have estimated the global yield of tomato wastes to be around 5.4–9.0 million tonnes, which are currently partially reused for animal feeding or by composting; however, the main fraction is disposed in landfills [5,6]. Rational strategies for valorizing this underexploited agricultural waste could be addressed since it has been reported to contain interesting amounts of oil and fatty acids [4,8], carotenoids [9,10,11], tocopherols [7], and polyphenols [12], among other biomolecules, which might find potential uses in added value applications.

Traditionally, mechanical processing or conventional extraction techniques using organic solvents have been employed to recover oil from plant seeds. However, microwave-assisted extraction (MAE) and supercritical fluid extraction (SFE) techniques have gained major importance in recent years, being a part of the green chemistry principles due to their reduced environmental impact combined with increased extraction yields and reduced extraction times and solvent consumption [13,14,15]. MAE is based on the ability of microwave irradiation to penetrate into certain materials and interact with polar components generating heat [16]. This mechanism allows selective heating from the inside of materials depending on their dielectric constant, generating internal pressure, which contributes to cell wall breakage, easy solvent penetration and target compounds dissolution. Consequently, mass and heat transfer phenomena work in the same direction leading to enhanced extraction performances [17]. In the case of SFE, supercritical fluids are used which exhibit liquid- and gas-like properties at temperature and pressure conditions above their critical point [18]. Then, mass transfer is enhanced by the relatively low viscosity and high diffusivity of supercritical fluids while extraction time is reduced. Carbon dioxide is one of the most common solvents used in SFE, due to its low critical temperature and pressure, avoiding thermolabile compounds degradation. Moreover, CO_2_ is environmentally friendly, highly available, non-toxic, easy to remove and generally recognized as safe by the FDA, which makes it especially useful for food and natural product applications [19,20]. Due to its low polarity, SC-CO_2_ has been used for lipophilic substances extraction, such as fatty acids, lipids, and carotenoids; however, co-solvents such as ethanol, methanol or acetonitrile could be added to favor highly polar compounds extraction.

SFE has been used for seed oil extraction from different natural sources and agricultural wastes, including tomato (*Lycopersicon esculentum* L.) seeds and pomace [21,22], *Pterodon emarginatus* Vogel seeds [23], *Passiflora* seeds [24], *Citrullus colocynthis* L. seeds [25], *Chenopodium quinoa* seeds [26], and *Echium vulgare* seeds [27]. Similarly, MAE has been employed to extract oil and lipids from *Gossypium arboreum* seeds [28], *Xanthoceras sorbifolia* Bunge seeds [29], *Viburnum opulus* seeds [30], and *Hura crepitans* seeds [31]. However, to the best of our knowledge, neither has MAE been used to isolate tomato seed oil (TSO) nor a comparative study on TSO recovery from TS using MAE and SFE has been reported so far. In this context, this work aimed at optimizing MAE and SFE-CO_2_ procedures for the extraction of TSO from tomato seeds to increase the added value of this agricultural waste. The optimal extraction conditions for maximizing both TSO recovery and FAMEs content by gas chromatography (GC) were determined by RSM. In the case of MAE, the influence of three extraction variables, such as extraction temperature, time, and solvent volume was studied, whereas pressure, extraction temperature, and flow rate were evaluated for SFE-CO_2_. Moreover, structural properties, tocopherols content, and antioxidant activity of optimized TSO were also studied by Fourier transform infrared spectroscopy (FTIR), high performance liquid chromatography (HPLC-DAD), and UV-vis spectroscopy, respectively.

## 2. Materials and Methods

### 2.1. Raw Material and Reagents

Raw dried tomato seed wastes were provided by SSICA (Parma, Italy) and they were ground into a powder using a ZM 200 high-speed rotatory mill (Restch, Hann, Germany). Particles passing through a 1.0 mm sieve were used for fatty acids extraction to ensure the homogeneity of the sample. All chemicals used in this work were of analytical grade and they were purchased from Sigma-Aldrich (Madrid, Spain).

### 2.2. Microwave-Assisted Extraction (MAE)

MAE was performed using a Milestone flexiWAVE^TM^ microwave oven (Milestone srl, Bergamo, Italy) in the open vessel configuration by heating the solvent under reflux. A mixture of ethyl acetate:ethanol (2:1, *v*/*v*) was selected as extraction solvent according to polarity requirements for microwave heating and previous optimization tests. This mixture of food grade solvents represents a greener alternative compared to other organic solvents commonly used for fatty acids extraction, such as n-hexane, dichloromethane, and chloroform [32]. According to preliminary tests (data not shown), the tomato seed powder (TS) amount was fixed at 3.0 g. During the extraction process, samples were stirred at 400 rpm to enhance heat and mass transfer. For the optimization of fatty acids extraction, different combinations of temperature (T), time (t), and solvent volume (V) were used according to Table 1. The obtained extracts were cooled to room temperature and centrifuged at 5300 rpm for 10 min. Then, the solid residue was washed with the extraction solvent and discarded. Afterwards, the supernatant was pooled with the washing solvent and tomato seed oil (TSO) was obtained after eliminating the extraction solvent using a rotary evaporator (R-300, Büchi Labortechnik AG, Switzerland).

### 2.3. Supercritical Fluid Extraction (SFE-CO_2_)

SFE-CO_2_ was carried out using a Process 100 bench scale extraction unit (Superfast, Thar Process, Pittsburgh, PA, USA). The system consisted of an extractor with an internal volume of 100 mL, a separator, a heat exchanger, and a carbon dioxide recycle storage tank. According to preliminary tests (data not shown), tomato seed powder (TS) amount was fixed at 20.0 g. During the extraction process, TS was introduced into the cylindrical extractor and the pressure was controlled using a back-pressure regulator. For the optimization of TSO extraction, different combinations of pressure (P), extraction temperature (T), and CO_2_ flow rate (F) were used. The obtained extracts were separated from CO_2_ by pressure reduction and they were collected in the separator. Finally, free CO_2_ extracts were cooled and CO_2_ was recycled into the CO_2_ storage tank.

### 2.4. Box-Behnken Experimental Designs (BBD)

**Figure 1:**
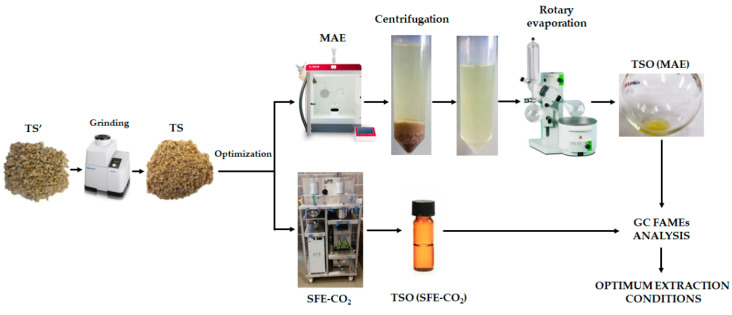
General scheme of fatty acids extraction optimization by microwave-assisted extraction (MAE) and supercritical fluid extraction (SFE-CO_2_).

MAE and SFE-CO_2_ optimization of fatty acids from TS was performed by using Box-Behnken experimental designs (BBD) and response surface methodology (RSM) in order to determine the optimum extraction conditions which maximized the studied responses. In total, 15 runs and 3 central points were used and all experimental runs were performed randomly. The range of the studied variables for each technique was selected based on preliminary experiments, equipment limitations and previously reported information in the literature [22,31,33,34,35]. For MAE, the effect of three independent variables (extraction temperature, extraction time and solvent volume) was studied. Extraction yield and concentration of four major fatty acids (palmitic acid (C16:0), stearic acid (C18:0), oleic acid (C18:1), and linoleic acid (C18:2)) were selected as response variables. In the case of SFE-CO_2_, the effect of three independent variables (pressure, extraction temperature, and CO_2_ flow rate) was evaluated, and extraction yield was selected as main response variable for comparison purposes. A general scheme of MAE and SFE-CO_2_ processes optimization is shown in Figure 1.

Multiple linear regression analysis of experimental data was performed in order to fit an empirical second-order polynomial model for each response variable, according to the following equation:

(1) $$Y={\;\beta }_{0}+\sum^{\;}\beta_{i}X_{i}+\sum^{\;}\beta_{i}X_{i}^{2}+\sum^{\;}\sum^{\;}\beta_{\mathrm{ij}}X_{i}X_{j},$$

where Y represents the predicted response variable, X_i_ and X_j_ represent the independent variables, β_0_ is a constant coefficient, and β_i_, β_ii_, and β_ij_ are the regression coefficients of linear, quadratic, and interaction effect terms, respectively. Lack of fit test and coefficient of determination (R^2^) were used to assess the adequacy of the fitted models to predict the experimental data. Statistical significance of model parameters was determined at the 5% probability level (α = 0.05) and ANOVA was used to analyze the principal effects and interactions of independent variables on the studied responses.

### 2.5. Tomato Seed Oil Characterization

#### 2.5.1. Extraction Yield

The extraction yield was gravimetrically determined by using the following equation:

(2) $$\mathrm{Yield}\;\left(\%\right)=100\;\frac{m_{\mathrm{TSO}}}{m_{\mathrm{TS}}}$$

where m_TSO_ is the weight of tomato seed oil obtained after rotary evaporation and m_TS_ is the weight of dried tomato seed powder used for extraction.

#### 2.5.2. Fatty Acid Methyl Esters (FAMEs) Content by GC

Fatty acid methyl esters (FAMEs) were prepared by subjecting 0.1 g of TSO to a methylation process. Briefly, 4.0 mL of a 0.2 mol L^−1^ methanolic sodium methoxide solution were added and the mixture was heated under reflux until total dissolution. Then, the base excess was neutralized with 1.0 mol L^−1^ sulfuric acid diluted in methanol and the mixture was heated under reflux for 5 min. Once the methylation reaction was completed and the mixture was cooled down to room temperature, 16 mL of a saturated sodium chloride solution were added. Finally, 3.0 mL of isooctane were incorporated to the mixture and FAMEs were extracted after vortex stirring. The upper organic layer was collected and subjected to gas chromatography analysis to determine the FAMEs content.

The identification of main FAMEs was performed by gas chromatography-mass spectrometry (GC-MS) using an Agilent 5973 GC-MS (Agilent technologies, Santa Clara, CA, USA) equipped with a BPX70 capillary column (60 m × 0.25 mm × 0.25 µm). An initial column temperature of 120 °C followed by a linear gradient of 3 °C min^−1^ up to 245 °C (maintained for 15 min) were used, accounting for a total analysis time of 56.7 min. The transfer line was kept at 300 °C. Mass spectra were analyzed in the scanning mode (30–450 m/z) and compared to NIST database and analytical standards for FAMEs identification, by analysis of their mass fragmentation patterns. The quantitative analysis of FAMEs was performed by gas chromatography with flame ionization detection (GC-FID). An Agilent 7820A GC-FID (Agilent technologies, Santa Clara, CA, USA) equipped with a Teknokroma TR-CN100 capillary column (60 m × 0.25 mm × 0.2 µm) was used. The oven temperature was held at 175 °C for 15 min and then raised to 220 °C (held for 1 min) at 10 °C min^−1^, accounting for a total analysis time of 20.5 min. The FID detector temperature was 250 °C, and hydrogen and air flow rates were 30 and 400 mL min^−1^, respectively. Quantification was carried out using external calibration by plotting the area of the corresponding peaks against concentration. Each sample was analyzed in triplicate. For both techniques, the injection was performed at 250 °C and 1 μL of FAMEs or standard solutions was injected (split ratio of 20:1) using He (1.0 mL min^−1^) as carrier gas.

#### 2.5.3. Fourier Transform Infrared Spectroscopy (FTIR)

TSO obtained under optimum MAE conditions was characterized by Fourier transform infrared spectroscopy (FTIR). A Bruker Analitik IFS 66/S spectrometer (Ettlingen, Germany) was used and the FTIR spectrum was recorded from 4000 to 400 cm^−1^ with an average of 64 scans at 4 cm^−1^ resolution. This instrument was equipped with a KBr beam splitter and a DTGS detector. OPUS software (Version 3.1), also developed by Bruker Analitik, was used for the analysis of spectra. The attenuated total reflectance (ATR) mode was used using a Golden Gate accessory with diamond crystal.

#### 2.5.4. Tocopherols Content by HPLC-DAD

Total contents of α- and γ-tocopherols in TSO extract were determined by high-performance liquid chromatography (HPLC-DAD), as described by Gliszczyńska-Świgło et al. [36], with slight modifications. An Agilent 1260 Infinity Quaternary LC HPLC system (Agilent Technologies, Palo Alto, CA, USA) equipped with a diode array detector was used. A Teknokroma Brisa LC2 C_18_ column (150 mm × 4.6 mm × 5 µm) coupled to a Teknokroma TR-C-160-1 ODS guard column (10 × 3.2 mm) were used operating at 25 °C. The mobile phase was methanol:acetonitrile (50:50, *v*/*v*) with a flow rate of 1.4 mL min^−1^ under isocratic conditions and the detector wavelength was set at 292 nm. TSO and standards for calibration curves of γ-tocopherol (0.6–70.0 mg kg^−1^, R^2^ = 1.0000) and α-tocopherol (0.1–3.0 mg kg^−1^, R^2^ = 0.9985) were prepared using 2-propanol as solvent. All samples were passed through a 0.45 µm nylon membrane filter prior to HPLC analysis and 20 µL of sample or standards were injected. Triplicate runs were carried out for each sample and the results were expressed as mg kg_TS_^−1^.

#### 2.5.5. Antioxidant Activity by DPPH• Radical Scavenging Method

Antioxidant activity of TSO was determined by DPPH• scavenging assay following the experimental conditions described by Szabo et al. [6], with some modifications. Briefly, 50 mg of TSO extract were dissolved in 2 mL of 2-propanol. Then, 0.4 mL of this solution were mixed with 2.1 mL of a freshly prepared DPPH• solution (10^−4^ mol L^−1^ in ethanol). The mixture was vortexed and incubated at room temperature in the dark for 120 min. Then, absorbance was measured at a wavelength of 517 nm against a pure ethanol blank. Trolox in 2-propanol was used as reference standard for quantification (0–60 mg kg^−1^, R^2^ = 0.9996). Results were expressed as milligrams of trolox equivalents (TE) per gram of TS and TSO. Each extract was analyzed in triplicate.

### 2.6. Statistical Analysis

Statgraphics Centurion XVI (Statistical Graphics, Rockville, MD, USA) was used to generate and analyze the BBD results. The graphic analysis of the main effects and interactions between variables was used and the analysis of variance (ANOVA) was carried out. SPSS 15.0 (Chicago, IL, USA) was used to perform the statistical analysis of experimental data by one-way analysis of variance (ANOVA). Differences between average values were assessed based on the Tukey test at a confidence level of 95% (*p* < 0.05).

## 3. Results and Discussion

### 3.1. MAE Optimization

#### 3.1.1. Model Fitting and Analysis

**Figure 2:**
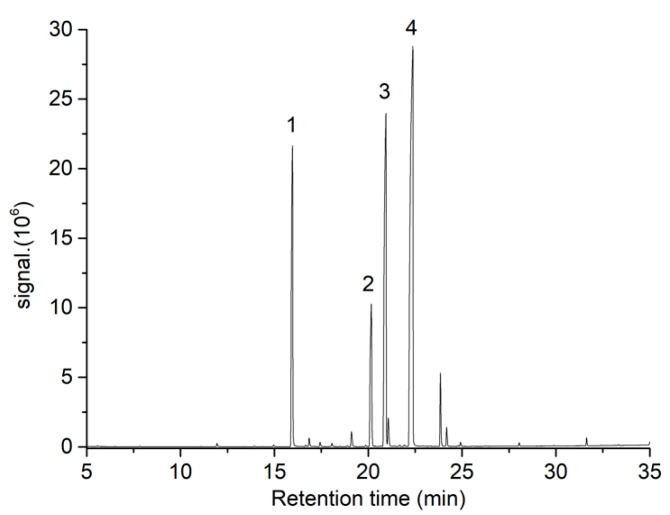
GC-MS chromatogram obtained from TSO by MAE. Peaks labeled as 1, 2, 3, and 4 correspond to methyl palmitate, methyl stearate, methyl oleate, and methyl linoleate, respectively.

The optimization of MAE conditions for oil recovery from a variety of plant seeds, such as *Cucurbita maxima* [37], *Viburnum opulus* [30], *Hura crepitans* [31], *Gossypium arboreum* [28], *Allanblackia parviflora* [38], and *Cannabis sativa* L. [39,40] has been recently reported. However, even though there might be some similarities compared to TS, these reported MAE conditions could not be directly extrapolated to other plant seeds due to their diverse nature and physicochemical composition, which might play a key role in microwave irradiation/sample interaction. Furthermore, it is well known that MAE could be influenced by several experimental factors; in consequence, process optimization should be carried out in each case study. In this work, the effect of extraction temperature, extraction time, and solvent volume on the MAE of fatty acids from TS was studied in terms of extraction yield and FAMEs content using a BBD with 15 independent runs, including 3 central points. The experimental conditions used and data obtained are shown in Table 1. Four major FAMEs were identified in TSO extracts by GC-MS (Figure 2): methyl palmitate, methyl stearate, methyl oleate, and methyl linoleate. These compounds were quantified by GC-FID obtaining satisfactory R^2^ values for calibration curves ranging from 0.991 to 0.999.

All studied responses were fitted to second-order mathematical models as a function of the independent factors by multiple regression analysis. The obtained mathematical models are presented in Equations (3)–(7):

Yield (g_TSO_ 100 g_TS_^−1^) = 16.20120 + 0.22707A − 0.04691B + 0.15141C − 0.00218A^2^ − 0.00161AB − 0.00017AC + 0.00108B^2^ + 0.00002BC − 0.00080C^2^ (3)

C16:0 (mg g_TS_^−1^) = −96.64160 + 1.50162A + 1.56288B + 1.94800C − 0.01212A^2^ + 0.00797AB − 0.01841AC − 0.01997B^2^ + 0.00683BC − 0.01402C^2^ (4)

C18:0 (mg g_TS_^−1^) = −37.41040 + 0.53138A + 0.59343B + 0.78512C − 0.00441A^2^ + 0.00366AB − 0.00703AC − 0.00787B^2^ + 0.00290BC − 0.00578C^2^ (5)

C18:1 (mg g_TS_^−1^) = −134.84800 + 1.97153A + 2.17416B + 2.83136C − 0.01568A^2^ + 0.01281AB − 0.02554AC − 0.02854B^2^ + 0.01031BC − 0.02068C^2^ (6)

C18:2 (mg g_TS_^−1^) = −345.27500 + 5.24847A + 5.58299B + 7.13366C − 0.04000A^2^ + 0.02852AB − 0.06528AC − 0.07144B^2^ + 0.02489BC − 0.05153C^2^ (7)

where A, B, and C represent extraction time, temperature, and solvent volume, respectively.

Analysis of variance (ANOVA) was carried out to study the effect of experimental factors on response variables as well as to evaluate the adequacy of the fitted models (Table 2). Non-significance of lack of fit test (*p* > 0.05) was observed, indicating that all mathematical models adequately fitted the experimental data. Moreover, acceptable R^2^ values were obtained for oil extraction yield, methyl palmitate, methyl stearate, methyl oleate, and methyl linoleate contents (0.8525, 0.8812, 0.8900, 0.8941, and 0.8832, respectively). These values indicated that more than 85% of variability could be explained by the fitted models for each response, highlighting an adequate level of correlation between the experimental data and predicted values.

#### 3.1.2. Effect of Extraction Variables on TSO Yield and FAMEs Content

The TSO extraction yield obtained by MAE ranged from 23.5 to 26.4 wt% under the tested experimental conditions (Table 1). No significant effects (*p* > 0.05) were observed for the studied independent variables on tomato seed extraction yield (Table 2), indicating that total lipids extraction was not influenced by the studied MAE experimental conditions. This behavior could be related to the possible co-extraction of several compounds at different extraction conditions, considering that the overall yield was gravimetrically calculated. However, Rezvankhah et al. [40] optimized the MAE of hempseed oil, reporting that both microwave power and extraction time showed significant linear and interaction effects on oil extraction yield. A similar study was conducted by Soroush et al. [39], who found significant linear and quadratic effects for solvent composition, microwave power, and irradiation time on hempseed extraction yield. Similarly, MAE optimization of oil extraction yield from *Hura crepitans* seeds showed significant effects for microwave power, extraction time, solvent composition, and solid-to-liquid ratio [31].

According to Naghdi et al. [41], total lipid recovery do not necessarily correlate with FAMEs recovery. The obtained contents of methyl palmitate, methyl stearate, methyl oleate, and methyl linoleate varied from 18.6 to 32.1, 7.3 to 12.7, 28.2 to 48.0, and 71.8 to 121.0 mg g_TS_^−1^, respectively. These results evidenced that FAMEs content in TSO was strongly influenced by MAE conditions. ANOVA analysis showed comparable significant effects (*p* < 0.05) for all FAMEs, these responses being similarly affected by extraction conditions. Consequently, it was expected that the same set of extraction conditions would maximize the four FAMEs responses, simultaneously. The four major FAMEs content was significantly affected (*p* < 0.05) by two linear positive (extraction time, A; and solvent volume, C) and two quadratic negative (temperature and solvent volume: B^2^ and C^2^, respectively) effects. Moreover, all FAMEs except methyl stearate were also significantly (*p* < 0.05) influenced by one interaction effect (AC). Overall, FAMEs content in TSO increased with increasing extraction time and solvent volume, while decreasing extraction temperature.

Extraction temperature caused a significant (*p* < 0.05) negative effect on the extraction of all FAMEs. It is well known that increasing extraction temperatures could enhance oil extraction efficiency, contributing to the structural damage caused in the oil-containing plant cells, combined with the decreased viscosity and increased permeation capacity of the solvent which enhance the release and solubility of target compounds into the extraction solvent [16,40]. However, excessive extraction temperatures could also lead to partial thermal decomposition of thermally sensitive oil components causing reduced MAE performances [39]. In fact, it has been reported that high temperatures may induce thermal damage, adversely affecting the oxidative state of lipids and consequently reducing their concentration in the extracted oil [40,42]. Therefore, milder extraction temperatures are usually used. Regarding extraction time, a positive linear effect was observed for all FAMEs contents. A longer contact between solvent and plant material contributes to its softening and swelling, weakening its wall integrity. However, longer extraction times may also expose plant tissues to excessive microwave irradiation, which could lead to undesirable thermal decomposition of FAMEs.

**Figure 3:**
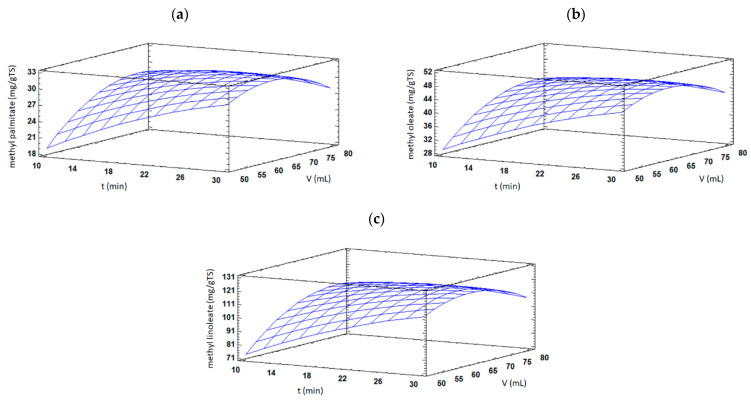
Response surface plots of significant interactions between extraction time vs. solvent volume on (**a**) methyl palmitate, (**b**) methyl oleate, and (**c**) methyl linoleate contents in TSO by MAE. In all cases, extraction temperature was fixed at its central value.

FAMEs content of TSO was also significantly (*p* < 0.05) affected by solvent volume, having positive linear and negative quadratic effects. Low amounts of extraction solvent might introduce mass transfer barriers, which limit the movement of compounds out from plant seeds. On the contrary, larger extraction volumes could enhance swelling and the rupture of the oil-containing seed cells promoting FAMEs extraction [16]. However, an excessive amount of solvent may produce a detrimental impact in microwave heating efficiency since a major fraction of microwave irradiation could be absorbed by the solvent instead of the plant material. Furthermore, the interaction effect between extraction time and solvent volume showed a significant (*p* < 0.05) negative effect on methyl palmitate, methyl oleate, and methyl linoleate contents. As shown in Figure 3a–c, an increase in extraction time at low solvent volume values resulted in increased FAMEs contents. However, increasing extraction time at high solvent volume values produced the opposite result and reduced FAME contents were found.

#### 3.1.3. Optimal Extraction Conditions and Verification Test

The experimental conditions which individually optimized each response variable as well as the optimum predicted value by the fitted models are shown in Table 3. As expected from the analysis of linear, quadratic and interaction effects of the extraction variables on the four major FAMEs content, very similar experimental conditions were found which maximized these responses. However, experimental conditions maximizing TSO extraction yield were slightly different. The combination of experimental factors, which simultaneously optimized all response variables, was determined by multi-response optimization using the desirability function. Optimum MAE conditions resulted in 29.0 min, 56.2 °C and 67.6 mL, with a desirability value of 1.00. At these conditions, predicted values determined at 95% level of probability in terms of TSO extraction yield, methyl palmitate, methyl stearate, methyl oleate, and methyl linoleate contents were 26.4 ± 1.2 wt%, 32.1 ± 3.8 mg g_TS_^−1^, 12.7 ± 1.5 mg g_TS_^−1^, 48.1 ± 5.2 mg g_TS_^−1^, and 121.2 ± 13.8 mg g_TS_^−1^, respectively, which were very similar to those obtained from single response optimization (Table 3).

Verification experiments were carried out under optimized extraction conditions, in triplicate, resulting in experimental responses in terms of TSO extraction yield, methyl palmitate, methyl stearate, methyl oleate, and methyl linoleate of 25.3 ± 0.3 wt%, 31.8 ± 1.0 mg g_TS_^−1^, 12.6 ± 0.4 mg g_TS_^−1^, 48.5 ± 1.4 mg g_TS_^−1^, and 121.7 ± 3.8 mg g_TS_^−1^, respectively. Thus, it was concluded that experimental results did not significantly differ (*p* > 0.05) from predicted values for all the studied responses. Moreover, relative standard deviations ranging from 1.1 to 3.2% were obtained for all analyzed responses, demonstrating the reproducibility of the developed TSO extraction method. In summary, the obtained models were reliable to predict the studied responses for the MAE of TSO, due to the high level of correlation between experimental data and predicted values.

### 3.2. SFE-CO_2_ Optimization

#### 3.2.1. Model Fitting and Analysis

The optimization of SFE-CO_2_ conditions for oil extraction from a variety of plant seeds, such as Passiflora [24], Punica granatum L. [43], Pterodon emarginatus Vogel [23], Silybum marianum [44], Citrullus colocynthis L. [25], and Echium vulgare [27], was recently reported. Similarly to MAE, these conditions could not be directly applied to other plant seeds and process optimization should be carried out in each case study. In this work, the effect of extraction temperature, pressure and flow rate on the SFE-CO_2_ of TSO from TS was studied using a BBD with 15 independent runs, including 3 central points. The characterization of TSO extracts was mainly based on extraction yield for comparison purposes with MAE. The experimental conditions and data obtained for the response variable are shown in Table 4.

TSO extraction yield was determined after SFE-CO_2_ extraction and linoleic acid content was quantified by GC-FID, accounting for approximately 50 wt% in all experiments (data not shown). According to the obtained results, a higher variability in extraction yield was found compared to the linoleic acid content. Thus, it was decided to use the extraction yield as the main response variable and it was fitted to a second-order mathematical model as a function of the independent factors by applying multiple regression analysis (Equation (8)):

Yield (g_TSO_ 100 g_TS_^−1^) = −19.62140 + 0.01366A + 0.67350B + 0.01230C + 0.00008A^2^ − 0.00044AB + 0.00022AC − 0.00629B^2^ + 0.00403BC − 0.00267C^2^, (8)

where A, B, and C represent pressure, extraction temperature, and flow rate, respectively.

ANOVA was carried out to study the effect of experimental factors on extraction yield and to evaluate the adequacy of the fitted model for SFE-CO_2_ (Table 5). A high R^2^ value (0.9489) was obtained for oil extraction yield, indicating that approximately 95% of variability could be explained by the model, highlighting a high level of correlation between experimental data and predicted values. Moreover, non-significance of the lack of fit test (*p* > 0.05) was observed, indicating that the mathematical model was reliable to fit the experimental data.

#### 3.2.2. Effect of Extraction Variables on TSO Extraction Yield and Optimal Extraction Conditions

The TSO extraction yield ranged from 1.7 to 16.8 wt% under all experiments performed (Table 4), evidencing that it was strongly influenced by SFE-CO_2_ conditions. TSO extraction yield was significantly affected (*p* < 0.05) by a linear effect of extraction pressure (A). Overall, TSO yield increased with increasing extraction pressure due to its positive effect. It is well known that SFE-CO_2_ efficiency could be enhanced by increasing extraction pressure, as it improves solvent density, as well as its ability to dissolve target compounds, leading to higher oil yields [27,45]. This density increase in supercritical CO_2_ contributes to decreasing the distance between the solvent molecules and lipophilic solutes, enhancing their interaction and leading to enhanced extraction yields and mass transfer rates [25]. However, an excessive extraction pressure could contribute to a decrease in supercritical CO_2_ diffusivity, limiting its ability to diffuse through the sample as well as to lead to sample compaction. In addition, economic feasibility of working at elevated pressure levels should be evaluated in each case study to determine whether the obtained extraction yield could balance energy consumption. Moreover, even though there was great variation within different extraction conditions, extraction temperature and CO_2_ flow rate showed no statistically significant effects (*p* > 0.05) on TSO extraction yield.

The combination of experimental conditions which optimized TSO yield resulted in 400 bar, 60.2 °C, and 64.6 g min^−1^. At these conditions, the optimum predicted value determined by the model at 95% level of probability in terms of extraction yield was 16.4 g TSO 100 g TS^−1^. Verification experiments were carried out under optimized SFE-CO_2_ conditions obtaining a TSO extraction yield of 16.9 g TSO 100 g TS^−1^. FAMEs composition in terms of methyl palmitate, methyl stearate, methyl oleate, and methyl linoleate resulted in experimental values of 16.5, 5.7, 20.3, and 50.2 wt%, respectively. The obtained results in this work are in agreement with those found by Eller et al. [21], who reported a yield of 17.3 wt% from tomato seeds oil by SFE-CO_2_. Moreover, our results were slightly higher compared to other authors, reporting TSO yields around 7.8 wt% for tomato waste (skins and seeds) and 12.5 wt% for tomato seeds using SC-CO_2_ methodologies [22,46]. In addition, comparable linoleic acid contents were obtained compared to those found by Durante et al. [46], Romano et al. [22], and Rozzi et al. [47], who extracted TSO from TS using SFE-CO_2_, reporting values of 44.8 wt%, 58.6 wt%, and 47.2 wt%, respectively.

### 3.3. Comparison of Extraction Techniques

Significantly higher (*p* < 0.05) TSO extraction yields were obtained in this work by MAE compared to SFE-CO_2_ at optimal extraction conditions (25.3 wt% and 16.9 wt%, respectively). Consequently, TSO obtained by MAE was selected for further characterization. However, similar fatty acids profiles were observed in terms of FAMEs composition (Table 6) for TSO at optimized extraction conditions. Thus, even though results suggested that MAE performance was better compared to SFE-CO_2_ in terms of TSO and fatty acids extraction yields, it should be highlighted that SFE-CO_2_ can be a potential technique for oil recovery since it provides some advantages, such as scalability and no need of further drying for eliminating residual organic solvents from solid residues after extraction. These characteristics could encourage its selection in biorefinery cascade approaches for integral wastes valorization. Similar conclusions were found by Teslić et al. [48], who carried out a comparative study between conventional and novel extraction techniques for wheat germ oil recovery. Even though these authors observed higher extraction yields and a substantially shortened extraction time by using MAE, SFE-CO_2_ was proposed as the most suitable extraction technique due to its advantages regarding environmental impact, energy consumption, and lack of organic solvents in recovering sample oil.

On the other hand, it is well known that physicochemical composition of vegetal materials, including tomato seeds, might vary according to cultivar, species, variety, soil characteristics, environmental conditions, harvesting time, and post-harvesting conditions, among other factors [5,49,50]. In consequence, a direct comparison of the obtained extraction yields with those found by other authors on TSO from tomato wastes might not be straightforward. Nevertheless, major advantages of the optimized MAE process developed in this work compared to conventional extraction techniques can be highlighted, such as enhanced extraction yields together with reducing extraction time, solvent consumption, and harmfulness. For instance, Szabo et al. [51] obtained TSO by using a simple solvent extraction process with n-hexane under continuous stirring for 2 h at room temperature, reporting a extraction yield of 19.1 ± 0.2 wt%. In another work, Azabou et al. [52] extracted TSO following a similar but longer procedure subjecting TS to conventional extraction with n-hexane for 4 h at room temperature, reporting an oil extraction yield of 17.15 wt%. Ouatmani et al. [53] reported a TSO extraction yield of 21.6 ± 1.5 wt% after Soxhlet extraction using n-hexane for 8 h. Botineştean et al. [54] used a solid-liquid semi-continuous Soxhlet extraction method for TSO recovery, reporting extraction yields ranging from 13.3 to 19.3 wt%, the extraction solvent used (diethyl ether or petroleum ether) directly influencing both extraction yield and relative FAMEs concentration values. Moreover, Giuffrè and Capocasale [49,55] extracted TSO from tomato seeds by using a Soxhlet-petroleum ether extraction process for 14 h, reporting extraction yields ranging from 19.8 to 23.4 wt%. Finally, Ozyurt et al. [56] optimized TSO extraction by means of cold press (CPE) and enzyme-assisted aqueous extraction (EAAE) procedures, reporting optimum oil extraction yields of 12.80 and 9.66 wt%, respectively. In conclusion, higher extraction yields were obtained in our study by MAE using also a greener alternative solvent combination (ethyl acetate:ethanol 2:1, *v*/*v*), also increasing the sustainability of the overall process.

**Table 6:** Comparison of fatty acid composition (wt%) of TSO obtained in this work and the reported results in literature.

Source	C16:0	C18:0	C18:1	C18:2	Σ	Method	Ref.
TS	14.83	5.87	22.59	56.71	100.0	MAE	This study
TS	16.50	5.70	20.30	50.20	92.7	SC-CO_2_	This study
TS	12.97	5.74	25.71	51.90	96.3	Soxhlet	[49]
TS	14.42	3.95	17.88	61.73	98.0	Stirring	[51]
TS	17.08	5.97	23.64	49.70	96.4	Stirring	[52]
TS	13.81	5.53	23.50	52.99	95.8	Soxhlet	[53]
TS	18.47	0.51	20.89	56.81	96.7	Semi-cont. Soxhlet	[54]
TS	7.76	9.28	24.95	56.59	98.6	CPE	[56]
TS	7.98	6.86	25.29	57.77	97.9	EAAE	[56]
TS	18.80	7.40	23.10	44.80	94.1	SC-CO_2_	[46]
Pomace	14.48	4.82	18.95	58.60	96.9	SC-CO_2_	[22]
TS	12.26	5.15	22.1	56.12	95.6	Soxhlet	[57]
TS	16.81	7.34	27.16	48.69	100.0	Vortex	[58]

TS: tomato seeds; CPE: cold press extraction; EAAE: enzyme-assisted aqueous extraction.

The composition of the fatty acid fraction obtained for both MAE and SFE-CO_2_ TSO extracts was in close agreement with the results reported by several authors, as is shown in Table 6. It is noteworthy that most cited examples do not sum up 100 wt% because the authors also quantified other minor FAMEs, such as myristic acid (C14:0), palmitoleic acid (C16:1), margaric acid (C17:0), linolenic acid (C18:3), arachidic acid (C20:0), gadoleic acid (C20:1), and behenic acid (C22:0), among others, which in all cases accounted for less than 6 wt%. Moreover, it could be noticed from Figure 2 that some of the aforementioned FAMEs might be also present in the obtained TSO. However, it was decided not to include them as response variables in the BBD due to their combined peak area represented only 4.6% of total peak areas.

Tomato seed oil has been reported to be an edible oil with high nutritional quality [4,5] according to its proportion of unsaturated fatty acids, accounting for around 80 wt% (Table 6). The quality and digestibility of edible vegetable oils are determined by the quantity and composition of their essential unsaturated fatty acids [59]. It was reported that linoleic acid may have favorable nutritional implications and beneficial physiological effects in preventing coronary heart and cancer diseases [52]. Moreover, it has been associated with several vital biological functions, such as antidiabetic, antiinflammatory, antiobesity, and antitumor activities [58]. Furthermore, it was reported that linoleic acid plays an important role in the healthy brain function, reproductive health, bone density, and cholesterol level regulation [54]. Regarding oleic acid, it is the most relevant monounsaturated fatty acid present in the human diet and it was also related to important bioactivities, such as antitumor, antiinflammatory, and cardioprotective functions [58]. Therefore, since linoleic and oleic acids are major components of TSO, it constitutes an interesting alternative source of fatty acids for functional foods, nutraceutical and pharmaceutical applications. In addition, the composition of TSO in terms of saturated and unsaturated fatty acids makes it a renewable energy source and promising fuel substitute for diesel engines in order to reduce the consumption of conventional petroleum-based products [57].

### 3.4. Characterization of TSO Obtained under Optimum MAE Conditions

#### 3.4.1. ATR-FTIR Analysis

**Figure 4:**
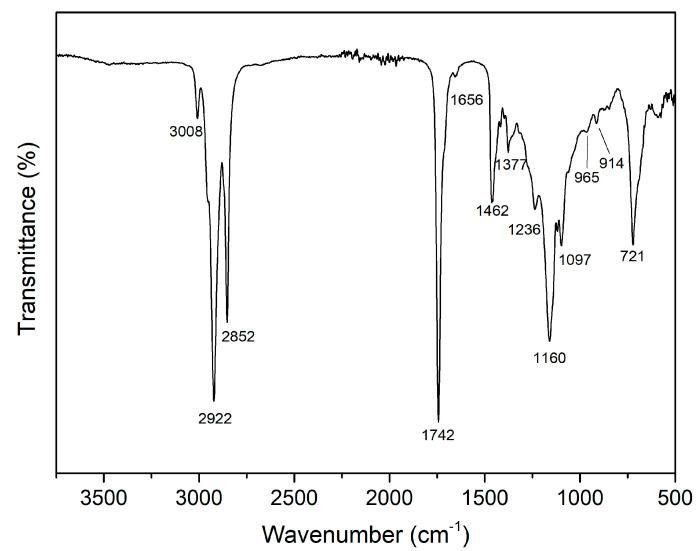
FTIR spectrum of TSO extract obtained under optimum MAE conditions.

A further characterization of the TSO extract obtained under optimum MAE conditions was performed using Fourier transform infrared spectroscopy (FTIR). The FTIR spectrum of tomato seed oil is shown in Figure 4, and it displayed the typical characteristic peaks reported for edible vegetable oils. Similar FTIR spectra were reported for seeds from tomato, pumpkin, pomegranate, and grape, as well as sunflower, sesame, and *Curcubita maxima* waste oils [60,61,62,63,64,65]. The observed characteristic peaks in the FTIR spectrum evidenced the presence of several functional groups. The peak appearing at 3008 cm^−1^ was assigned to the stretching vibration of cis C=CH. The peaks found at 2922 and 2852 cm^−1^ were related to asymmetric and symmetric stretching vibrations of methylene (-CH_2_), respectively. The carbonyl (C=O) stretching vibration was observed by a strong peak signal at 1742 cm^−1^, while the small peak appearing at 1656 cm^−1^ was associated to the C=C stretching vibration. The bending vibrations of methylene and methyl were observed at 1462 and 1377 cm^−1^, respectively. The peaks found at 1236, 1160, and 1097 cm^−1^ were assigned to C-O vibrations. The peaks showed at 965 and 914 cm^−1^ were linked to bending out of plane vibrations of –HC=CH–(trans), while the peak observed at 721 cm^−1^ corresponded to the bending out of plane vibrations of –HC=CH-(cis).

#### 3.4.2. Tocopherols Content and Antioxidant Activity

Vitamin E is a mixture of tocopherols and tocotrienols synthesized only by plants, with nuts, green leafy vegetables, and vegetable oils being the most usual natural sources. Tocopherols, which are also naturally present in plant seeds, have been extensively studied in recent years due to their well-known antiinflammatory, anticancer, and antioxidant activities [1,22]. In this study, γ-tocopherol was found as the dominant homologue (260.3 ± 0.6 mg kg_TS_^−1^), followed by a lower amount of α-tocopherol (6.53 ± 0.12 mg kg_TS_^−1^), accounting for 97.6 wt% and 2.4 wt%, respectively.

The tocopherols content found in TSO by MAE was in general agreement with those found by other authors. Westphal et al. [66] characterized seeds from two different tomato varieties and they reported γ-tocopherol concentrations ranging from 17.5 to 32.7 mg 100 g_TS_^−1^ (97–98 wt%), while α-tocopherol homologue accounted for only 0.56 to 0.68 mg 100 g_TS_^−1^ (2–3 wt%). In another work, da Silva and Neuza [59] isolated bioactive compounds from the lipid fraction of several agro-industrial wastes, including tomato seeds, reporting only the presence of the γ-tocopherol homologue, whereas α-tocopherol was not detected. Moreover, Eller et al. [21] and Durante et al. [46] recovered TSO from TS using SC-CO_2_ extraction, and they also found that vitamin E was predominantly composed of the gamma homologue, accounting for 94.6 wt% (181.7 mg kg_TS_^−1^) and 91.6 wt% (126.4 mg kg_TS_^−1^) of the total tocopherols content, respectively. Regarding α-tocopherol contents, 8.7 mg kg_TS_^−1^ and 11.5 mg kg_TS_^−1^ were found, respectively.

Other studies reported slightly different tocopherols composition in TSO. For instance, Müller et al. [67] found a fraction of 75 wt% of γ-tocopherol followed by approximately 17 wt% of α-tocopherol in the vitamin E content of TSO. Romano et al. [22] studied TSO recovered by conventional, liquid, and supercritical carbon dioxide extraction from tomato peels and pomace (seeds and peels), reporting γ-tocopherol and α-tocopherol contents ranging from 82.2 to 90 wt% and from 10 to 17.8 wt%, respectively. Furthermore, contents of 277.5 mg kg^−1^ and 30.8 mg kg^−1^ were found for γ- and α-tocopherol, respectively, from tomato pomace extracted with supercritical CO_2_. Alternatively, Ubeyitogullari and Ciftci [68] found 57.0 wt% and 40.2 wt% of γ-tocopherol and α-tocopherol, respectively, in TSO obtained from tomato pomace by-products using SC-CO_2_ extraction. In this case, the raw material used included a significant amount of tomato peels apart from tomato seeds, which might significantly influence the tocopherols composition of the recovered TSO. Moreover, Vági et al. [69] also studied the extraction of tocopherol from industrial tomato by-products using supercritical CO_2_ extraction, obtaining very differing tocopherol contents depending on the source used. In particular, one of the TSO samples contained 83.8 to 90.0 wt% of γ-tocopherol followed by 8.2 to 14.7 wt% of the α-tocopherol homologue. However, one tomato pomace sample showed the highest α-tocopherol content, accounting for 68.4 to 70.4 wt%, followed by the γ-tocopherol homologues (26.7 to 28.8 wt%). The authors attributed these dissimilarities to different cultivars as well as differences in the distribution of seed and skin fractions in the studied tomato pomace samples.

Even though the content and composition of vitamin E might widely vary according to sample characteristics and the extraction methods used, γ-tocopherol has been shown to be mainly predominant in vegetable seed oils. It has been reported that the variation in tocopherols content of TSO is highly dependent on processing conditions, raw material characteristics, and cultivar, among other factors [56]. In addition, it is known that tocopherols could act as chain breakers in free radical chain reactions enhancing the stability of oil during storage. Moreover, Ozyurt et al. [56] reported that γ-tocopherol degrades into two effective antioxidant dimers, whereas α-tocopherol leads to the formation of products with low or no antioxidant activity in TSO samples. Then, the presence of a high γ-tocopherol content in the TSO MAE extracts was promising in terms of possessing antioxidant activity.

The antioxidant activity of TSO was demonstrated by the DPPH• radical scavenging assay, which has been widely used to test the ability of compounds to act as free radical scavengers or hydrogen donors, as well as to evaluate the antioxidant activity of food samples, including several types of vegetable oils [30,37,38,56,70]. In this study, TSO exhibited high antioxidant activity, accounting for 0.437 ± 0.004 mg_TE_ g_TS_^−1^ (1.657 ± 0.031 mg_TE_ g_TSO_^−1^). The antioxidant activity of SFE-CO_2_ extracts was also determined, obtaining lower DPPH• scavenging values (0.151 ± 0.001 mg_TE_ g_TS_^−1^ and 0.941 ± 0.001 mg_TE_ g_TSO_^−1^). Similar findings were reported by other authors from TSO samples using different tomato varieties, wastes (i.e., seeds, peels, pomace), and processing, extraction, and analysis techniques. Giuffrè et al. [50] studied the antioxidant activity of TSO obtained by mechanical extraction, reporting a strong antioxidant activity in all TSO samples with values ranging from 71 to 88% of DPPH• radical inhibition after 30 min of incubation. Similar results were observed by Shao et al. [71], who recovered TSO from TS by conventional extraction using n-hexane, obtaining DPPH• scavenging activities ranging from 72 to 76%, showing TSO higher antioxidant activity compared to other vegetable oils. In another study, Ozyurt et al. [56] evaluated the antioxidant activity properties of TSO obtained by cold press and enzyme-assisted aqueous extraction procedures, reporting DPPH• radical scavenging inhibition values of 6.2% and 3.2%, respectively. These authors suggested that the antioxidant activity of TSO was directly related to its polyunsaturated fatty acids, tocopherols, and phenolics contents. These compounds were proposed to act as hydrogen donors or free radical acceptors, which could inhibit the chain reaction of oxidation and its antioxidant capacity could be determined by the decolorization of the DPPH• dissolution.

In summary, due to its interesting tocopherols content and antioxidant activity, TSO constitutes an interesting source of natural antioxidant compounds, which might contribute to replace synthetic antioxidants due to their possible adverse effects on human health [72]. Moreover, TSO might find potential uses in human nutrition, functional food development and cosmetic applications.

## 4. Conclusions

In this work, a comparative study using microwave-assisted extraction (MAE) and supercritical fluid extraction (SFE-CO_2_) for the recovery of lipids and bioactive compounds from tomato seed by-products was carried out for the first time. The combined effects of main extraction parameters for both techniques were studied on tomato seed oil (TSO) and fatty acids (palmitic acid, stearic acid, oleic acid, and linoleic acid) extraction yields and optimized by using response surface methodologies. Higher TSO extraction yields and antioxidant activity (DPPH• radical scavenging) were obtained by MAE compared to SFE-CO_2_, showing TSO extracts a high γ-tocopherol content. The developed MAE methodology could be a promising green and efficient approach for the valorization of tomato seed by-products with potential applications in functional food and nutrition fields, contributing to the circular economy by minimizing food waste and environmental impact issues in modern industries. Regarding SFE-CO_2_, it has a great potential for industrial scaling up and integral TS valorization in biorefinery cascade approaches, since solid residues do not require further drying steps to eliminate residual organic solvents after extraction, reducing process steps and costs.

## Figures and Tables

**Table 1:** BBD matrix and response values obtained for TSO extracts by MAE.

Experimental Design				Response Variables				
Run	t (min)	T (°C)	V (mL)	Yield (wt%)	C16:0 (mg g_TS_^−1^) *	C18:0 (mg g_TS_^−1^) *	C18:1 (mg g_TS_^−1^) *	C18:2 (mg g_TS_^−1^) *
1	30	40	65	25.2	26.5 ± 1.0	10.3 ± 0.5	39.9 ± 1.6	101.1 ± 3.9
2	30	55	50	24.3	27.8 ± 1.6	11.0 ± 0.7	42.1 ± 2.7	106.1 ± 6.3
3	10	55	50	23.9	19.1 ± 0.1	7.6 ± 0.1	29.2 ± 0.1	74.1 ± 0.2
4	30	55	80	25.4	28.1 ± 0.6	11.1 ± 0.4	42.7 ± 1.3	107.4 ± 3.2
5	30	70	65	26.2	30.7 ± 1.2	12.2 ± 0.4	46.5 ± 1.7	117.4 ± 4.2
6	20	40	80	25.4	24.4 ± 0.2	9.5 ± 0.2	37.0 ± 0.7	93.9 ± 1.6
7	20	55	65	24.6	32.1 ± 1.2	12.7 ± 0.5	48.0 ± 1.8	121.0 ± 4.5
8	20	70	80	26.4	23.9 ± 1.2	9.5 ± 0.5	36.6 ± 1.9	92.1 ± 4.6
9	10	70	65	25.4	21.2 ± 0.1	8.4 ± 0.1	32.3 ± 0.1	82.4 ± 0.2
10	20	55	65	24.8	29.8 ± 1.2	11.8 ± 0.5	44.8 ± 1.7	112.9 ± 4.1
11	10	40	65	23.5	21.7 ± 1.5	8.8 ± 0.6	33.4 ± 2.3	83.2 ± 5.4
12	10	55	80	25.0	30.4 ± 2.1	11.9 ± 0.8	45.1 ± 3.0	114.5 ± 8.3
13	20	70	50	24.8	18.6 ± 0.9	7.3 ± 0.4	28.2 ± 1.6	71.8 ± 3.7
14	20	55	65	25.8	30.2 ± 1.3	11.9 ± 0.6	45.2 ± 2.0	114.4 ± 5.1
15	20	40	50	23.8	25.3 ± 1.4	10.0 ± 0.6	37.9 ± 2.1	96.0 ± 5.3

t: extraction time; T: extraction temperature; V: solvent volume. * Mean ± SD, *n* = 3.

**Table 2:** ANOVA results for response surface quadratic models of TS extraction by MAE.

Source	Sum of Squares	Df	Mean Square	F-Value	*p*-Value
**Yield**					
A	1.28	1	1.28	3.05	0.2230
B	2.97	1	2.97	7.07	0.1171
C	3.56	1	3.56	8.48	0.1005
AA	0.18	1	0.18	0.42	0.5841
AB	0.23	1	0.23	0.56	0.5331
AC	0.00	1	0.00	0.01	0.9445
BB	0.22	1	0.22	0.52	0.5468
BC	0.00	1	0.00	0.00	0.9907
CC	0.12	1	0.12	0.29	0.6453
Lack-of-fit	0.65	3	0.22	0.51	0.7128
Pure error	0.84	2	0.42		
Total (corr.)	10.09	14			
R^2^	0.8525				
Adj R^2^	0.5870				
**Methyl palmitate (C16:0)**					
A	53.42	1	53.42	36.76	0.0261 *
B	1.62	1	1.62	1.11	0.4021
C	31.43	1	31.43	21.63	0.0433 *
AA	5.42	1	5.42	3.73	0.1931
AB	5.72	1	5.72	3.94	0.1857
AC	30.51	1	30.51	21.00	0.0445 *
BB	74.52	1	74.52	51.27	0.0190 *
BC	9.45	1	9.45	6.50	0.1255
CC	36.77	1	36.77	25.30	0.0373 *
Lack-of-fit	29.14	3	9.71	6.68	0.1329
Pure error	2.91	2	1.45		
Total (corr.)	269.70	14			
R^2^	0.8812				
Adj R^2^	0.6673				
**Methyl stearate (C18:0)**					
A	7.85	1	7.85	31.91	0.0299 *
B	0.21	1	0.21	0.85	0.4529
C	4.89	1	4.89	19.87	0.0468 *
AA	0.72	1	0.72	2.92	0.2295
AB	1.20	1	1.20	4.89	0.1575
AC	4.45	1	4.45	18.07	0.0511
BB	11.58	1	11.58	47.04	0.0206 *
BC	1.70	1	1.70	6.91	0.1194
CC	6.25	1	6.25	25.39	0.0372 *
Lack-of-fit	4.09	3	1.36	5.54	0.1566
Pure error	0.49	2	0.25		
Total (corr.)	41.70	14			
R^2^	0.8900				
Adj R^2^	0.6920				
**Methyl oleate (C18:1)**					
A	121.12	1	121.12	40.52	0.0238 *
B	2.67	1	2.67	0.90	0.4438
C	71.66	1	71.66	23.97	0.0393 *
AA	9.07	1	9.07	3.04	0.2236
AB	14.77	1	14.77	4.94	0.1563
AC	58.70	1	58.70	19.64	0.0473 *
BB	152.24	1	152.24	50.93	0.0191 *
BC	21.53	1	21.53	7.20	0.1153
CC	79.93	1	79.93	26.74	0.0354 *
Lack-of-fit	54.35	3	18.12	6.06	0.1449
Pure error	5.98	2	2.99		
Total (corr.)	569.52	14			
R^2^	0.8941				
Adj R^2^	0.7034				
**Methyl linoleate (C18:2)**					
A	758.10	1	758.10	41.26	0.0234 *
B	13.74	1	13.74	0.75	0.4783
C	446.88	1	446.88	24.32	0.0387 *
AA	59.07	1	59.07	3.22	0.2148
AB	73.18	1	73.18	3.98	0.1841
AC	383.57	1	383.57	20.88	0.0447 *
BB	953.97	1	953.97	51.92	0.0187 *
BC	125.42	1	125.42	6.83	0.1206
CC	496.28	1	496.28	27.01	0.0351 *
Lack-of-fit	382.33	3	127.44	6.94	0.1286
Pure error	36.74	2	18.37		
Total (corr.)	3587.66	14			
R^2^	0.8832				
Adj R^2^	0.6729				

A: extraction time; B: extraction temperature; C: solvent volume. * Significant effect at *p* < 0.05.

**Table 3:** Single response optimized MAE conditions and predicted values to maximize TSO extraction yield and FAMEs content.

Response	t (min)	T (°C)	V (mL)	Predicted Value
Yield ^a^	23.0	70.0	80.0	26.4
C16:0 ^b^	30.0	56.0	63.4	32.2
C18:0 ^b^	30.0	56.4	63.8	12.7
C18:1 ^b^	30.0	56.4	64.0	48.4
C18:2 ^b^	30.0	56.2	63.8	122.1

t: extraction time; T: extraction temperature; V: solvent volume. ^a^ TSO extraction yield expressed as g TSO 100 g TS^−1^; ^b^ methyl palmitate (C16:0), methyl stearate (C18:0), methyl oleate (C18:1), and methyl linoleate (C18:2) contents expressed as mg g TS^−1^.

**Table 4:** BBD matrix and response values obtained for TSO extracts by SFE-CO_2_.

	Experimental Design			Response Variable
Run	P (bar)	T (°C)	F (g min^−1^)	Yield (wt%)
1	400	60	75	16.8
2	400	60	25	13.8
3	250	40	25	4.1
4	100	60	25	2.1
5	100	80	50	4.3
6	250	60	50	8.5
7	250	40	75	3.0
8	400	80	50	10.4
9	250	60	50	9.8
10	100	60	75	1.8
11	250	80	25	1.7
12	400	40	50	13.9
13	250	80	75	8.7
14	250	60	50	7.5
15	100	40	50	2.5

P: pressure; T: extraction temperature; F: flow rate; dCO_2_: CO_2_ density.

**Table 5:** ANOVA results for response surface quadratic models of TS extraction by SFE-CO_2_.

Source	Sum of Squares	Df	Mean Square	F-Value	*p*-Value
**Yield**					
A	244.43	1	244.43	187.39	0.0053 *
B	0.30	1	0.30	0.23	0.6768
C	9.37	1	9.37	7.19	0.1155
AA	11.02	1	11.02	8.45	0.1008
AB	6.97	1	6.97	5.34	0.1470
AC	2.82	1	2.82	2.16	0.2791
BB	23.40	1	23.40	17.94	0.0515
BC	16.24	1	16.24	12.45	0.0718
CC	10.27	1	10.27	7.87	0.1070
Lack-of-fit	15.02	3	5.01	3.84	0.2136
Pure error	2.61	2	1.30		
Total (corr.)	344.56	14			
R^2^	0.9489				
Adj R^2^	0.8568				

A: pressure; B: extraction temperature; C: flow rate. * Significant effect at *p* < 0.05.

## Data Availability

Data are contained within the article.

## References

[1] Gómez-Romero M., Arráez-Román D., Segura-Carretero A., Fernández-Gutiérrez A. (2007). Analytical Determination of Antioxidants in Tomato: Typical Components of the Mediterranean Diet. J. Sep. Sci..

[2] Pinela J., Prieto M.A., Barreiro M.F., Carvalho A.M., Oliveira M.B.P.P., Vázquez J.A., Ferreira I.C.F.R. (2016). Optimization of Microwave-Assisted Extraction of Hydrophilic and Lipophilic Antioxidants from a Surplus Tomato Crop by Response Surface Methodology. Food Bioprod. Process..

[3] Minoggio M., Bramati L., Simonetti P., Gardana C., Iemoli L., Santangelo E., Mauri P.L., Spigno P., Soressi G.P., Pietta P.G. (2003). Polyphenol Pattern and Antioxidant Activity of Different Tomato Lines and Cultivars. Ann. Nutr. Metab..

[4] Kumar M., Chandran D., Tomar M., Bhuyan D.J., Grasso S., Sá A.G.A., Carciofi B.A.M., Radha, Dhumal S., Singh S. (2022). Valorization Potential of Tomato (*Solanum lycopersicum* L.) Seed: Nutraceutical Quality, Food Properties, Safety Aspects, and Application as a Health-Promoting Ingredient in Foods. Horticulturae.

[5] Lu Z., Wang J., Gao R., Ye F., Zhao G. (2019). Sustainable Valorisation of Tomato Pomace: A Comprehensive Review. Trends Food Sci. Technol..

[6] Szabo K., Diaconeasa Z., Cătoi A.F., Vodnar D.C. (2019). Screening of Ten Tomato Varieties Processing Waste for Bioactive Components and Their Related Antioxidant and Antimicrobial Activities. Antioxidants.

[7] Laranjeira T., Costa A., Faria-Silva C., Ribeiro D., de Oliveira J.M.P.F., Simões S., Ascenso A., Ferreira de Oliveira J.M.P., Simões S., Ascenso A. (2022). Sustainable Valorization of Tomato by-products to Obtain Bioactive Compounds: Their Potential in Inflammation and Cancer Management. Molecules.

[8] Benítez J.J., Castillo P.M., del Río J.C., León-Camacho M., Domínguez E., Heredia A., Guzmán-Puyol S., Athanassiou A., Heredia-Guerrero J.A. (2018). Valorization of Tomato Processing by-products: Fatty Acid Extraction and Production of Bio-Based Materials. Materials.

[9] Casa M., Miccio M., De Feo G., Paulillo A., Chirone R., Paulillo D., Lettieri P., Chirone R. (2021). A Brief Overview on Valorization of Industrial Tomato by-products Using the Biorefinery Cascade Approach. Detritus.

[10] Szabo K., Cătoi A.-F., Vodnar D.C. (2018). Bioactive Compounds Extracted from Tomato Processing by-products as a Source of Valuable Nutrients. Plant Foods Hum. Nutr..

[11] Madia V.N., De Vita D., Ialongo D., Tudino V., De Leo A., Scipione L., Di Santo R., Costi R., Messore A. (2021). Recent Advances in Recovery of Lycopene from Tomato Waste: A Potent Antioxidant with Endless Benefits. Molecules.

[12] Domínguez R., Gullón P., Pateiro M., Munekata P.E.S., Zhang W., Lorenzo J.M. (2020). Tomato as Potential Source of Natural Additives for Meat Industry. A Review. Antioxidants.

[13] Ferreira I.J.B., Alexandre E.M.C., Saraiva J.A., Pintado M. (2022). Green Emerging Extraction Technologies to Obtain High-Quality Vegetable Oils from Nuts: A Review. Innov. Food Sci. Emerg. Technol..

[14] Rani H., Sharma S., Bala M. (2021). Technologies for Extraction of Oil from Oilseeds and Other Plant Sources in Retrospect and Prospects: A Review. J. Food Process Eng..

[15] Kultys E., Kurek M.A. (2022). Green Extraction of Carotenoids from Fruit and Vegetable Byproducts: A Review. Molecules.

[16] Chan C.-H., Yusoff R., Ngoh G.-C., Kung F.W.-L. (2011). Microwave-Assisted Extractions of Active Ingredients from Plants. J. Chromatogr. A.

[17] Veggi P.C., Martínez J., Meireles M.A.A., Chemat F., Cravotto G. (2013). Fundamentals of Microwave Extraction. Microwave-Assisted Extraction for Bioactive Compounds: Theory and Practice.

[18] Picot-allain C., Mahomoodally M.F., Ak G., Zengin G. (2021). Conventional versus Green Extraction Techniques—A Comparative Perspective. Curr. Opin. Food Sci..

[19] Strati I.F., Oreopoulou V. (2014). Recovery of Carotenoids from Tomato Processing by-Products—A Review. Food Res. Int..

[20] Aniceto J.P.S., Rodrigues V.H., Portugal I., Silva C.M. (2021). Valorization of Tomato Residues by Supercritical Fluid Extraction. Processes.

[21] Eller F.J., Moser J.K., Kenar J.A., Taylor S.L. (2010). Extraction and Analysis of Tomato Seed Oil. J. Am. Oil Chem. Soc..

[22] Romano R., Aiello A., Pizzolongo F., Rispoli A., De Luca L., Masi P. (2020). Characterisation of Oleoresins Extracted from Tomato Waste by Liquid and Supercritical Carbon Dioxide. Int. J. Food Sci. Technol..

[23] Chañi-Paucar L.O., Johner J.C.F., Zabot G.L., Meireles M.A.A. (2022). Technical and Economic Evaluation of Supercritical CO_2_ Extraction of Oil from Sucupira Branca Seeds. J. Supercrit. Fluids.

[24] Liu S., Yang F., Zhang C., Ji H., Hong P., Deng C. (2009). Optimization of Process Parameters for Supercritical Carbon Dioxide Extraction of Passiflora Seed Oil by Response Surface Methodology. J. Supercrit. Fluids.

[25] Chouaibi M., Rigane K., Ferrari G. (2020). Extraction of Citrullus Colocynthis L. Seed Oil by Supercritical Carbon Dioxide Process Using Response Surface Methodology (RSM) and Artificial Neural Network (ANN) Approaches. Ind. Crops Prod..

[26] Daud N.M., Putra N.R., Jamaludin R., Md Norodin N.S., Sarkawi N.S., Hamzah M.H.S., Mohd Nasir H., Abang Zaidel D.N., Che Yunus M.A., Md Salleh L. (2022). Valorisation of Plant Seed as Natural Bioactive Compounds by Various Extraction Methods: A Review. Trends Food Sci. Technol..

[27] Bilgiç-Keleş S., Şahin-Yeşilçubuk N., Barla-Demirkoz A., Karakaş M. (2019). Response Surface Optimization and Modelling for Supercritical Carbon Dioxide Extraction of Echium Vulgare Seed Oil. J. Supercrit. Fluids.

[28] Taghvaei M., Jafari S.M., Assadpoor E., Nowrouzieh S., Alishah O. (2014). Optimization of Microwave-Assisted Extraction of Cottonseed Oil and Evaluation of Its Oxidative Stability and Physicochemical Properties. Food Chem..

[29] Zhang D.-Y., Yao X.-H., Luo M., Zhao C., Fu Y.-J. (2016). Optimization of Negative Pressure Cavitation–Microwave Assisted Extraction of Yellow Horn Seed Oil and Its Application on the Biodiesel Production. Fuel.

[30] Dursun Capar T., Dedebas T., Yalcin H., Ekici L. (2021). Extraction Method Affects Seed Oil Yield, Composition, and Antioxidant Properties of European Cranberrybush (Viburnum Opulus). Ind. Crops Prod..

[31] Ibrahim A.P., Omilakin R.O., Betiku E. (2019). Optimization of Microwave-Assisted Solvent Extraction of Non-Edible Sandbox (Hura Crepitans) Seed Oil: A Potential Biodiesel Feedstock. Renew. Energy.

[32] Prat D., Wells A., Hayler J., Sneddon H., McElroy C.R., Abou-Shehada S., Dunn P.J. (2016). CHEM21 Selection Guide of Classical- and Less Classical-Solvents. Green Chem..

[33] Solaberrieta I., Jiménez A., Garrigós M.C. (2022). Valorization of Aloe Vera Skin by-Products to Obtain Bioactive Compounds by Microwave-Assisted Extraction: Antioxidant Activity and Chemical Composition. Antioxidants.

[34] Vallecilla-Yepez L., Ciftci O.N. (2018). Increasing Cis-Lycopene Content of the Oleoresin from Tomato Processing Byproducts Using Supercritical Carbon Dioxide. LWT.

[35] Lisichkov K., Kuvendziev S., Lisichkov B. (2011). Isolation of Tomato Seed Oil from Tomato Waste by Application of Supercritical Fluid CO_2_ Extraction. Qual. Life.

[36] Gliszczyńska-Świgło A., Sikorska E. (2004). Simple Reversed-Phase Liquid Chromatography Method for Determination of Tocopherols in Edible Plant Oils. J. Chromatogr. A.

[37] Jiao J., Li Z.-G., Gai Q.-Y., Li X.-J., Wei F.-Y., Fu Y.-J., Ma W. (2014). Microwave-Assisted Aqueous Enzymatic Extraction of Oil from Pumpkin Seeds and Evaluation of Its Physicochemical Properties, Fatty Acid Compositions and Antioxidant Activities. Food Chem..

[38] Quaisie J., Ma H., Golly M.K., Tuly J.A., Amaglo N.K., Jiaqi Z. (2021). Effect of Ultrasound-Microwave Irradiation Hybrid Technique on Extraction, Physicochemical, Antioxidative, and Structural Properties of Stearic Acid-Rich Allanblackia Parviflora Seed Oil. Chem. Pap..

[39] Soroush D.R., Solaimanimehr S., Azizkhani M., Kenari R.E., Dehghan B., Mohammadi G., Sadeghi E. (2021). Optimization of Microwave-Assisted Solvent Extraction of Hemp (*Cannabis sativa* L.) Seed Oil Using RSM: Evaluation of Oil Quality. J. Food Meas. Charact..

[40] Rezvankhah A., Emam-Djomeh Z., Safari M., Askari G., Salami M. (2019). Microwave-Assisted Extraction of Hempseed Oil: Studying and Comparing of Fatty Acid Composition, Antioxidant Activity, Physiochemical and Thermal Properties with Soxhlet Extraction. J. Food Sci. Technol..

[41] Naghdi F.G., Thomas-Hall S.R., Durairatnam R., Pratt S., Schenk P.M. (2014). Comparative Effects of Biomass Pre-Treatments for Direct and Indirect Transesterification to Enhance Microalgal Lipid Recovery. Front. Energy Res..

[42] Mushtaq A., Roobab U., Denoya G.I., Inam-Ur-Raheem M., Gullón B., Lorenzo J.M., Barba F.J., Zeng X.A., Wali A., Aadil R.M. (2020). Advances in Green Processing of Seed Oils Using Ultrasound-Assisted Extraction: A Review. J. Food Process. Preserv..

[43] Liu G., Xu X., Hao Q., Gao Y. (2009). Supercritical CO2 Extraction Optimization of Pomegranate (*Punica granatum* L.) Seed Oil Using Response Surface Methodology. LWT-Food Sci. Technol..

[44] Lukic I., Milovanovic S., Pantic M., Srbljak I., Djuric A., Tadic V., Tyśkiewicz K. (2022). Separation of High-Value Extracts from Silybum Marianum Seeds: Influence of Extraction Technique and Storage on Composition and Bioactivity. LWT.

[45] Pavlić B., Pezo L., Marić B., Tukuljac L.P., Zeković Z., Solarov M.B., Teslić N. (2020). Supercritical Fluid Extraction of Raspberry Seed Oil: Experiments and Modelling. J. Supercrit. Fluids.

[46] Durante M., Montefusco A., Marrese P.P., Soccio M., Pastore D., Piro G., Mita G., Lenucci M.S. (2017). Seeds of Pomegranate, Tomato and Grapes: An Underestimated Source of Natural Bioactive Molecules and Antioxidants from Agri-Food by-Products. J. Food Compos. Anal..

[47] Rozzi N.L., Singh R.K., Vierling R.A., Watkins B.A. (2002). Supercritical Fluid Extraction of Lycopene from Tomato Processing byproducts. J. Agric. Food Chem..

[48] Teslić N., Bojanić N., Čolović D., Fišteš A., Rakić D., Solarov M.B., Zeković Z., Pavlić B. (2020). Conventional versus Novel Extraction Techniques for Wheat Germ Oil Recovery: Multi-Response Optimization of Supercritical Fluid Extraction. Sep. Sci. Technol..

[49] Giuffrè A.M., Capocasale M. (2016). Physicochemical Composition of Tomato Seed Oil for an Edible Use: The Effect of Cultivar. Int. Food Res. J..

[50] Giuffrè A.M., Capocasale M., Zappia C. (2017). Tomato Seed Oil for Edible Use: Cold Break, Hot Break, and Harvest Year Effects. J. Food Process. Preserv..

[51] Szabo K., Dulf F.V., Teleky B.-E., Eleni P., Boukouvalas C., Krokida M., Kapsalis N., Rusu A.V., Socol C.T., Vodnar D.C. (2021). Evaluation of the Bioactive Compounds Found in Tomato Seed Oil and Tomato Peels Influenced by Industrial Heat Treatments. Foods.

[52] Azabou S., Louati I., Ben Taheur F., Nasri M., Mechichi T. (2020). Towards Sustainable Management of Tomato Pomace through the Recovery of Valuable Compounds and Sequential Production of Low-Cost Biosorbent. Environ. Sci. Pollut. Res..

[53] Ouatmani T., Haddadi-Guemghar H., Boulekbache-Makhlouf L., Mehidi-Terki D., Maouche A., Madani K. (2022). A Sustainable Valorization of Industrial Tomato Seeds (Cv Rio Grande): Sequential Recovery of a Valuable Oil and Optimized Extraction of Antioxidants by Microwaves. J. Food Process. Preserv..

[54] Botineştean C., Gruia A.T., Jianu I. (2015). Utilization of Seeds from Tomato Processing Wastes as Raw Material for Oil Production. J. Mater. Cycles Waste Manag..

[55] Giuffrè A.M., Capocasale M. (2015). Policosanol in Tomato (*Solanum lycopersicum* L.) Seed Oil: The Effect of Cultivar. J. Oleo Sci..

[56] Ozyurt V.H., Çakaloğlu B., Otles S. (2021). Optimization of Cold Press and Enzymatic-Assisted Aqueous Oil Extraction from Tomato Seed by Response Surface Methodology: Effect on Quality Characteristics. J. Food Process. Preserv..

[57] Giannelos P.N., Sxizas S., Lois E., Zannikos F., Anastopoulos G. (2005). Physical, Chemical and Fuel Related Properties of Tomato Seed Oil for Evaluating Its Direct Use in Diesel Engines. Ind. Crops Prod..

[58] Li Y., Yuan F., Wu Y., Zhang Y., Gao B., Yu L. (2020). Triacylglycerols and Fatty Acid Compositions of Cucumber, Tomato, Pumpkin, and Carrot Seed Oils by Ultra-Performance Convergence Chromatography Combined with Quadrupole Time-of-Flight Mass Spectrometry. Foods.

[59] Da Silva A.C., Jorge N. (2014). Bioactive Compounds of the Lipid Fractions of Agro-Industrial Waste. Food Res. Int..

[60] Irnawati, Riyanto S., Martono S., Rohman A. (2019). The Employment of FTIR Spectroscopy and Chemometrics for Authentication of Pumpkin Seed Oil from Sesame Oil. Food Res..

[61] Riyanta A.B., Riyanto S., Lukitaningsih E., Rohman A. (2019). The Employment of Fourier Transform Infrared Spectroscopy (FTIR) and Chemometrics for Analysis of Candlenut Oil in Binary Mixture with Grape Seed Oil. Food Res..

[62] Riyanta A.B., Riyanto S., Lukitaningsih E., Rohman A. (2020). Analysis of Sunflower Oil in Ternary Mixture with Grapeseed Oil and Candlenut Oil in the Ternary Mixture System Using FTIR Spectroscopy and Chemometrics. Food Res..

[63] Uncu O., Napiórkowska A., Szajna T.K., Ozen B. (2020). Evaluation of Three Spectroscopic Techniques in Determination of Adulteration of Cold Pressed Pomegranate Seed Oils. Microchem. J..

[64] Ibrahim K.A., Abu-sbeih K.A., Al-Trawneh I., Bourghli L. (2014). Preparation and Characterization of Alkyd Resins of Jordan Valley Tomato Oil. J. Polym. Environ..

[65] Hundie K.B. (2022). Optimization of Biodiesel Production Parameters from Cucurbita Maxima Waste Oil Using Microwave Assisted via Box-Behnken Design Approach. J. Chem..

[66] Westphal A., Bauerfeind J., Rohrer C., Ernawita, Böhm V. (2014). Analytical Characterisation of the Seeds of Two Tomato Varieties as a Basis for Recycling of Waste Materials in the Food Industry. Eur. Food Res. Technol..

[67] Müller L., Catalano A., Simone R., Cittadini A., Fröhlich K., Böhm V., Palozza P. (2013). Antioxidant Capacity of Tomato Seed Oil in Solution and Its Redox Properties in Cultured Macrophages. J. Agric. Food Chem..

[68] Ubeyitogullari A., Ciftci O.N. (2022). Enhancing the Bioaccessibility of Lycopene from Tomato Processing Byproducts via Supercritical Carbon Dioxide Extraction. Curr. Res. Food Sci..

[69] Vági E., Simándi B., Vásárhelyiné K.P., Daood H., Kéry Á., Doleschall F., Nagy B. (2007). Supercritical Carbon Dioxide Extraction of Carotenoids, Tocopherols and Sitosterols from Industrial Tomato by-Products. J. Supercrit. Fluids.

[70] Kiralan M., Özkan G., Bayrak A., Ramadan M.F. (2014). Physicochemical Properties and Stability of Black Cumin (Nigella Sativa) Seed Oil as Affected by Different Extraction Methods. Ind. Crops Prod..

[71] Shao D., Atungulu G.G., Pan Z., Yue T., Zhang A., Li X. (2012). Study of Optimal Extraction Conditions for Achieving High Yield and Antioxidant Activity of Tomato Seed Oil. J. Food Sci..

[72] Blasi F., Cossignani L. (2020). An Overview of Natural Extracts with Antioxidant Activity for the Improvement of the Oxidative Stability and Shelf Life of Edible Oils. Processes.

